# LPS aggravates lung inflammation induced by RSV by promoting the ERK-MMP-12 signaling pathway in mice

**DOI:** 10.1186/s12931-020-01453-6

**Published:** 2020-07-21

**Authors:** Shenglin Chen, Jun Xie, Keting Zhao, Luo Ren, Yu Deng, Xiaohong Xie, Shiyi Chen, Hongmei Xu, Xiaoru Long, Enmei Liu

**Affiliations:** 1 Pediatric Research Institute, Children’s Hospital of Chongqing Medical University, Chongqing, China; 2 China International Science and Technology Cooperation Base of Child development and Critical Disorders, Chongqing, China; 3 Chongqing Key Laboratory of Pediatrics, Chongqing, China; 4 Chongqing Key Laboratory of Child Infection and Immunity, Chongqing, China; 5 Chongqing Medical University, Chongqing, China; 6 Center for Clinical Molecular Medicine, Chongqing Stem Cell Therapy Technology Research Center, Children’s Hospital of Chongqing Medical University, Chongqing, China; 7 Lijia respiratory Department, Ministry of Education Key Laboratory of Child Development and Disorders; National Clinical Research Center for Child Health and Disorders; China International Science and Technology Cooperation base of Child development and Critical Disorders, Children’s Hospital of Chongqing Medical University, Chongqing, 400014 P. R. China; 8 Department of Infection, Children’s Hospital of Chongqing Medical University, Chongqing Key Laboratory of Child Infection and Immunity, Chongqing, 400014 P. R. China

**Keywords:** RSV, LPS, MMP-12, Lung inflammation, AHR

## Abstract

**Background:**

RSV can lead to persistent airway inflammation and airway hyperresponsiveness (AHR), and is intimately associated with childhood recurrent wheezing and asthma, but the underlying mechanisms remain unclear. Lipopolysaccharide (LPS) is also implicated in the onset and exacerbation of asthma. However, whether inhalation of LPS can boost airway inflammation induced by RSV is not clear. In this study, we utilized an LPS- and RSV-superinfected mouse model to explore underlying pathogenesis.

**Methods:**

Mice were infected with RSV on day 0 and inoculated with LPS from day 35 to day 41, samples were collected on day 42. Inflammatory cells, lung histopathology and AHR were measured. Cytokines were detected by ELISA and ERK, JNK, p38 was determined by western blot. MMP408, PD98059, SP600125 and SB203580 were used to inhibit MMP-12, ERK, JNK and p38 respectively.

**Results:**

LPS exposure superimposed on RSV-infected lungs could lead to more vigorous cellular influx, lung structures damage, augmented AHR and higher MMP-12 levels. Inhibition of MMP-12 or ERK signaling pathway in vivo both diminished LPS-driven airway inflammation and AHR.

**Conclusions:**

Exposure to LPS in RSV-infected mice is associated with enhanced increases in ERK-MMP-12 expression that translates into increased lung inflammation and AHR. These findings contribute novel information to the field investigating the onset of post-RSV bronchiolitis recurrent wheezing as a result of LPS exposure.

## Background

Respiratory syncytial virus (RSV) is the most frequent cause of lower respiratory infection (LRTI) in infants worldwide. An estimated 30–70% of infants develop bronchiolitis upon primary RSV infection, 1–3% of whom are hospitalized [1, 2]. Moreover, studies conducted in different parts of the world and with different study designs, have shown that RSV LRTI / bronchiolitis in early life is associated with up to a five-fold increase in risk of developing recurrent wheezing and asthma later in childhood [3]. A birth cohort study that has been ongoing for almost four decades showed that early life RSV LRTI is associated with a persistently low lung function trajectory that represents an important pathway to COPD [4]. Despite the risks of RSV infection being well accepted, the precise mechanisms leading to the onset of these chronic airway diseases are poorly understood.

Co-infection of bacteria and viruses had a synergistic effect, leading to more severe disease and hospitalization [5]. It’s reported that RSV-infected infants are colonized with pathogenic bacteria and have a higher proportion of Gram-negative bacterial colonization compared to healthy age-matched controls [6]. In addition to bacterial co-infection, environmental pollution is also an adverse risk to asthma exacerbation [7]. Lipopolysaccharide (LPS) is a major compound of wall for Gem-negative bacteria, and also acts as a significant immunostimulatory component of air pollution. Both epidemiological studies and animal experiments have demonstrated that exposure to LPS can sensitize or exacerbate COPD and asthma by promoting Th2 responses [8]. We have identified that a Th2 cytokines-dominant airway microenvironment similar to asthma was triggered during the later stage of RSV infection [9]. However, it’s not clear whether inhalation of LPS might boost RSV-associated airway inflammation and AHR by increasing Th2 responses.

In addition to Th2 cytokines, our preliminary results have also demonstrated that matrix metalloproteinases (MMPs), especially MMP-12 are important in RSV pathogenesis [10, 11]. MMP-12 is mainly secreted by macrophages. It regulates the turnover of extracellular matrix (ECM) and is widely involved in the pathogenesis of chronic airway diseases including asthma and COPD, and also delays viral clearance by affecting IFN-α [12]. In vitro studies stated that LPS could induce MMP-12 in mouse liver macrophages [13] and human bronchial epithelial cells [14]. However, whether LPS exposure can affect RSV-associated MMP-12 overproduction is also not clear.

LPS is recognized by Toll-like receptor 4 (TLR4) and MD-2 on host innate immune cells and can signal to activate the MAP kinase proteins (MAPK), finally leading to the increase of multi-inflammatory mediators and co-stimulatory molecules. Among the three MAPK proteins, the ERK1/2 kinase induces gene expression responsible for mucin production via promoting MUC5AC in inflammatory cells resulting in mucus production in asthmatic model. JNK kinase has been reported to be associated with T cell activation and maturation, whereas p38 plays an important role in inducing expression of pro-inflammatory mediators IL-1β, TNF-α, IL-8, IL-6, and IL-3, especially in the macrophages [15]. MMP-12 was induced in the LPS-treated bronchial epithelial cells, which was decreased by ERK inhibition [14]. Moreover, cigarette smoke could activate ERK pathway that mediated the DC-induced Th2 polarization [16]. In this pre-clinical study, we utilized an LPS- and RSV-superinfected mouse model to explore whether LPS can exaggerate airway disorders by boosting the MAPK-Th2 cytokines / MMP-12 pathway.

## Methods

### Animal

Adult 6-week-old female BALB/c mice were purchased from the Animal Laboratory of Chongqing Medical University and housed in individually filtered cages. All experiments involving animals were in accordance with the Guide for the Care and Use of Laboratory Animals and approved by the Institutional Animal Care and Committee (IACUC), which is accredited by the Association for Assessment and Accreditation of Laboratory Animal Care International, China and Experimental Animal Committee of the Chongqing Medical University (license numbers: SCXK (Yu) 2012–0001 and SYXK (Yu) 2012–0001).

### Virus preparation

RSV A2 (ATCC, VR-1540) strain was propagated in Hep-2 cells (ATCC) with Dulbecco’s Modified Eagle’s Medium (DMEM; GIBCO, California, USA) plus 5% fetal bovine serum (FBS, GIBCO). Viral titer was determined by plaque assay. The supernatants of uninfected Hep-2 cells were generated under the same condition.

### LPS preparation

LPS (*Escherichia coli* 055:B5, Sigma), was assayed using the limulus hemocyanin agglutination assay. Ambient environmental LPS exposure concentrations have been reported up to 10,000 endotoxin units (EU)/m^3^ although ambient levels are usually below 1000 EU/m^3^. 1 EU is equivalent to approximately 0.1 ng LPS and so 1000 EU is equivalent to 0.1 mg LPS. Our LPS challenge method with 10 ng was therefore within the level bounds of exposure that would usually occur in the environment. Certainly, the LPS doses that we used are in accordance with previous publications [17].

### Inoculation procedure

Mice were anaesthetized with isoflurane and held upright before intranasal inoculation. Mice were divided into four groups:

- The control group: Mice were mock-infected with 100 μl cell supernatant on day 0 and then inoculated with 50 μl of PBS every 2 days from day 35 to day 41.
- The LPS group: Mice were mock-infected with 100 μl cell supernatant on day 0 and then inoculated with 10 μg LPS dissolved in 50 μl PBS every 2 days from day 35 to day 41.
- The RSV group: Mice were infected with RSV (1.8*10^7^ PFU in 100 μl of virus supernatant) on day 0 and then inoculated with 50 μl of PBS every 2 days from day 35 to day 41 post RSV infection.
- The RSV + LPS group: Mice were infected with RSV on day 0 and then inoculated with 10 μg LPS dissolved in 50 μl PBS every 2 days from day 35 to day 41 post RSV infection.

Disease parameters were assessed on day 42.

### Whole lung lavage

Following euthanasia, the trachea was cannulated and bronchoalveolar lavage fluid (BALF) was collected for cytokine concentration measurement and inflammatory cell evaluation. The total number of cells was quantified by automated cell counter (Count Star, China). Cytospin slides were fixed and stained with DiffQuik (Baxter Healthcare Corp, Deerfield, Miami, FL) for leukocyte differential analysis.

### Flow cytometry analysis

The single cell suspensions of mouse lung tissue were prepared as described previously [9]. Samples were blocked with rat serum for 20 min, and then immunostained with antibody to mouse CD45, CD3, CD49b, CD19, F4/80, CD11c, CD11b, Ly6G, Ly6C or isotype control conjugated with PerCP-cy7, PerCP-cy5.5, PE, FITC or APC for 30 min on ice. The indicated antibodies were obtained from eBioscience (San Diego, CA) or BD Biosciences or Invitrogen. Next, stained samples were fixed with 1% Formaldehyde in FACS Staining Buffer and measured on a flow cytometer, FACSCalibur (BD Biosciences), which collected data on at least 5000/10,000 events.

### Lung histology

For histology studies, left-lung lobes from mice were removed, fixed in 10% formalin, cut into 5 μm sections, and stained with HE solution (Sigma, St. Louis, MO, USA). Images were captured under a Nikon Eclipse E200 microscope connected to a Nikon Coolpix 995 camera (Nikon, Tokyo, Japan).

### Airway hyper-responsiveness (AHR)

AHR was measured 24 h after the final LPS challenge by measuring the lung resistance (LR) using an invasive lung function test. Animals were anesthetized with pentobarbital (30 mg/kg, ip) and connected via a tracheostomy tube to a computer-controlled piston ventilator (flexiVent, Scireq). Subsequently, mice were exposed to aerosolized acetyl-β-methylcholine (Sigma-Aldrich, Saint Louis, MO, USA), at increasing doses: 0, 3.125, 6.25, 12.5, 25, and 50 mg/ml. At each dose, LR was calculated using the single-compartment model.

### Cytokine analysis

The levels of IL-4, IL-5, IL-13, IFN-γ, IL-10, IL-6, IL-17, IL-21, IL-1β, MMP-9 and MMP-12 in BALF were measured using an enzyme-linked immunosorbent assay (ELISA) with commercial kits (eBioscience, CA, USA)) according to the manufacturer’s instructions. Duplicate wells were run, and the mean values were reported.

### RNA extraction, reverse transcription, and quantitative PCR (qPCR)

Total RNA from mouse lung tissues was purified, and cDNA synthesis was performed using a PrimeScript RTReagent Kit according to manufacturer’s recommendations (Takara, Otsu, Japan). Quantitative PCR (qPCR) was performed using standard techniques [18]. GAPDH was used as endogenous controls. The primer sequences of GATA3 were 5′-CTCGGCCATTCGTACATGGAA-3' (forward) and 5'-GGATACCTCTGCACCGTAGC-3' (reverse); ID2 were 5'-GCATCCCACTATCGTCAGCC-3' (forward) and 5'-AAGGGAATTCAGATGCCTGCAA-3' (reverse); MMP-12 were 5'-CGATGTGGAGTGCCCGATGT-3' (forward) and 5′- AGTCTCCGTGAGCTCCAAATGC-3′ (reverse); and GAPDH were 5′-AGCAATGCCTCCTGCACCACCAAC-3′ (forward) and 5′-CCGGAGGGGCCATCCACAGTCT-3′ (reverse).

### Western blotting analysis

Total protein of mice lung tissues were obtained and the concentrations were determined as previously reported [11]. Samples were separated on an 8% SDS-PAGE gels, transferred onto PVDF membranes (Millipore, Billerica, MA), bathed in blocking buffer for 1 h at room temperature, and then incubated overnight at 4 °C with primary antibody of ERK (Santa Cruz Biotechnology, 1:500), p-ERK (Santa Cruz Biotechnology, 1:500), JNK (Santa Cruz Biotechnology, 1:500), p-JNK (Santa Cruz Biotechnology, 1:500), p38 (Santa Cruz Biotechnology, 1:1000), p-p38 (Santa Cruz Biotechnology, 1:1000) or GAPDH (Santa Cruz Biotechnology, 1:3000) respectively. An alkaline phosphatase-conjugated goat anti-mouse antibody (MultiSciences, China, 1:5000) was used to detect the presence of the respective protein bands. Densitometry of bands from Western blots was done by ImageJ2x 2.1.4.7 (Wayne Rasband, National Institutes of Health, USA), and the densities of the ERK, p-ERK, JNK, p-JNK, p38, p-p38 proteins relative to GAPDH were measured.

### MMP-12 inhibition

Mice were treated with MMP408, a potent and specific MMP-12 inhibitor (CALBIOCHEM, EMD Chemicals, Inc. San Diego, CA 92121) at 5 mg/kg intragastrically twice a day consecutively from day 35 to day 41 post RSV infection. The control animals received sterile PBS similarly.

### MAPK pathway inhibition

To assess the effects of ERK, JNK, and p38 pathway on the airway disorders induced by LPS in our model, where indicated, mice were treated with the specific inhibitors of ERK (PD98059, invivogen, 10 mg/kg) or JNK (SP600125, invivogen, 20 mg/kg) or p38 (SB203580, invivogen, 5 mg/kg) respectively. The inhibitor was solubilized in 2% dimethyl sulfoxide (DMSO; Sigma-Aldrich, St. Louis, MO, USA). Treatments with inhibitors or DMSO alone were given intraperitoneally 1 h before and 2 h after the first LPS inoculation on day 35, and once a day successively from day 36 to day 41.

### Statistical analysis

All statistical tests were performed using Prism GraphPad Software (La Jolla, CA), and the results are expressed as the mean ± sem. Two-way ANOVA with Bonferroni post-tests were used to compare the differences among multiple groups to AHR. Analysis of variance (ANOVA) was used to determine the differences between all groups to other indices. Data lacking normal distribution were evaluated using the nonparametric Kruskal-Wallis test, followed by Dunn’s multiple comparison. Differences were considered to be significant for *p*-values less than 0.05.

## Results

### LPS increases lung inflammation and AHR during the later stage of RSV infection

**Fig. 1:**
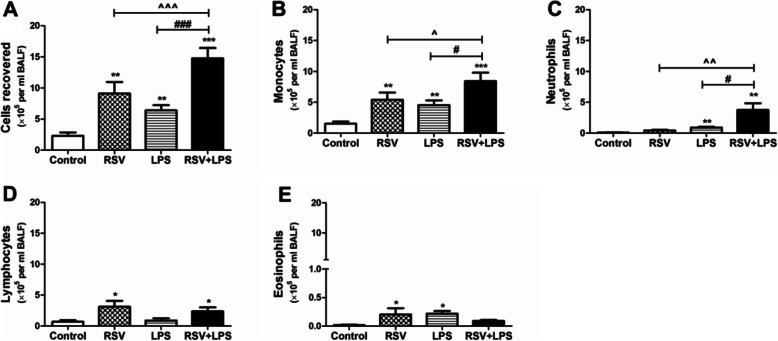
Inflammatory cells in bronchoalveolar lavage fluid (BALF). Mice were infected or mock-infected with RSV or cell supernatant on day 0 and then inoculated with LPS as described in the materials and methods part. BALF was collected and inflammatory cells were counted. **a**: Total inflammatory cells. **b**: Monocytes. **c**: Neutrophils. **d**: Lymphocytes. **e**: Eosinophils. Graphs are represented as the mean ± sem. Data are representative of two independent experiments performed on 8 animals per group. *, *p* < 0.05, **, *p* < 0.01, ***, *p* < 0.001, shown comparing the control group with the other groups; #, *p* < 0.05, ###, *p* < 0.001, shown comparing the RSV + LPS group with the LPS group; ^, *p* < 0.05, ^^, *p* < 0.01, ^^^, *p* < 0.001, shown comparing the RSV + LPS group with the RSV group

**Fig. 2:**
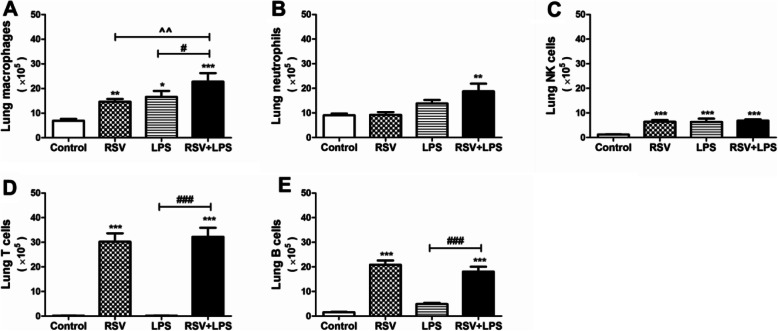
Inflammatory cells in lung tissues. The single cell suspensions of mouse lung tissue were prepared and detected with flow cytometry. **a**: Macrophages. **b**: Neutrophils. **c**: NK cells. **d**: T cells. **e**: B cells. Data are representative of two independent experiments performed on 8 animals per group. *, *p* < 0.05, **, *p* < 0.01, ***, *p* < 0.001, shown comparing the control group with the other groups; #, *p* < 0.05, ###, *p* < 0.001, shown comparing the RSV + LPS group with the LPS group; ^^, *p* < 0.01, shown comparing the RSV + LPS group with the RSV group

Mice were infected with RSV on day 0 and then inoculated with LPS as described in the materials and methods section. As shown in Fig. 1, inflammatory cells (A), monocytes (B), neutrophils (C) in BALF were dramatically aggravated in the RSV + LPS group as compared to the control group, the RSV group and the LPS group (all *P* < 0.05). Lung tissue flow cytometric analysis revealed that macrophages (Fig. 2a) were dramatically aggravated in the RSV + LPS group as compared to the control group, the RSV group and the LPS group. Neutrophils (Fig. 2b), NK cells (Fig. 2c), T cells (Fig. 2d) and B cells (Fig. 2e) were all significantly increased in the RSV + LPS group as compared to the control group (all *P* < 0.05), but showed no significant difference versus the RSV group and the LPS group.

**Fig. 3:**
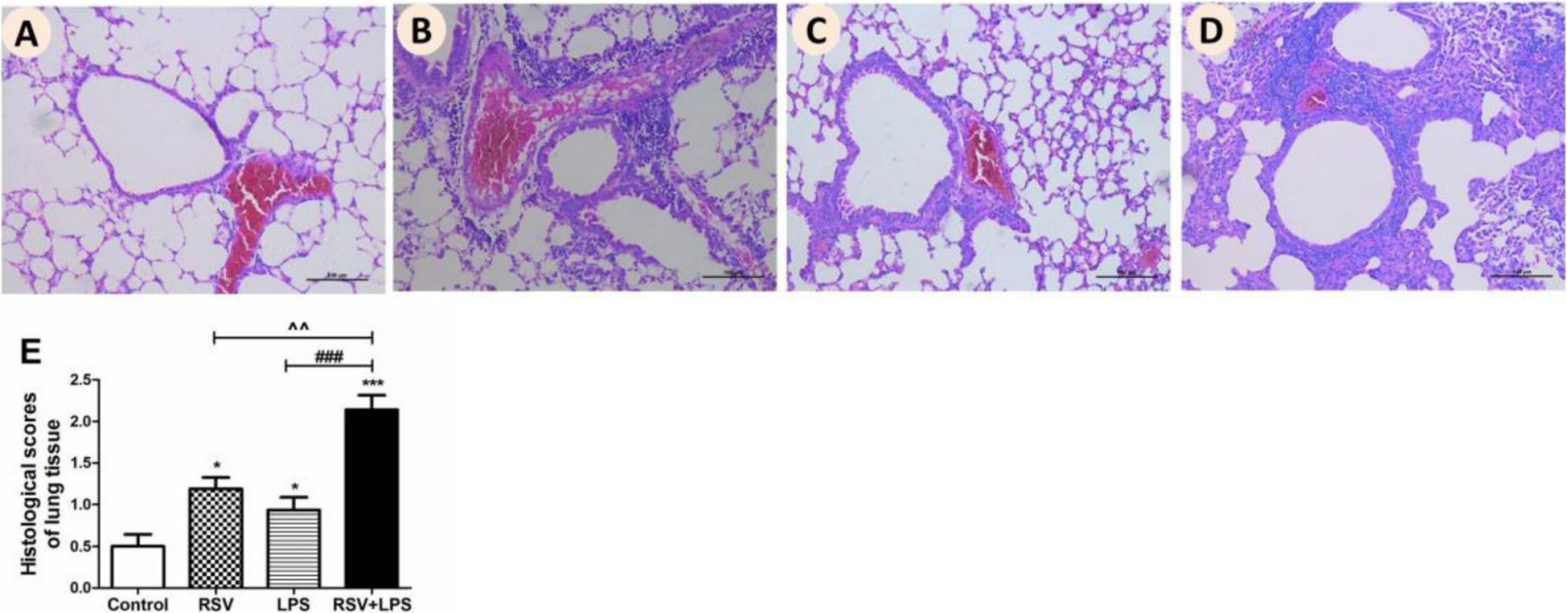
Pathological damage of lung tissues. Lungs were harvested on day 42 post-RSV infection, stained with hematoxylin-and-eosin (HE), and scored for levels of inflammation as described in the Materials and Methods section. Representative HE staining of mouse lung tissue sections (original Magnification × 200) were shown. **a**: control group; **b**: RSV group; **c**: LPS group; **d**: RSV + LPS group. Histological scores (HPS) were assessed (**e**). Graphs are represented as the mean ± sem. Data are representative of two independent experiments performed on 6 animals per group. *, *p* < 0.05, ***, *p* < 0.001, shown comparing the control group with the other groups; ###, *p* < 0.001, shown comparing the RSV + LPS group with the LPS group; ^^, *p* < 0.01, shown comparing the RSV + LPS group with the RSV group

Next, lung tissue HE staining and histological scores (HPS) were performed to estimate pathological injury. As shown in Fig. 3a, no pulmonary hyper-cellularity or other pathological characteristics were observed in the control group. However, in the other mice groups, a mass of inflammatory cells infiltrated in the interstitial, peribronchiolar and perivascular compartments, with partial destruction of regular tissue structures (Fig. 3b-d). HPS (Fig. 3e) was similar to the morphological changes, which was markedly increased in the RSV + LPS group compared with the other three groups (all *P* < 0.05).

**Fig. 4:**
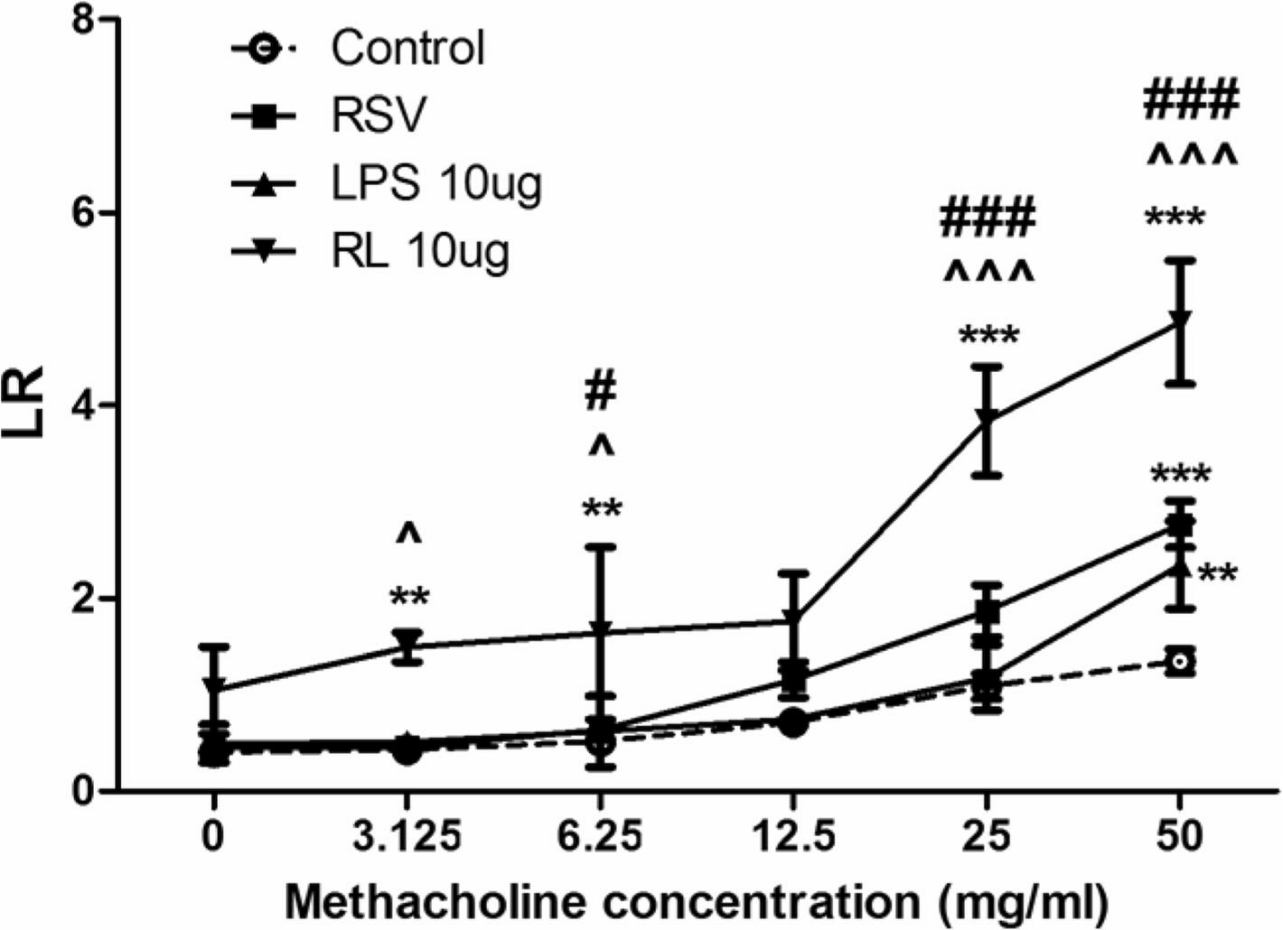
Airway hyperresponsiveness (AHR). AHR was assessed by measuring the lung resistance (LR) to increasing doses of methacholine. Graphs are represented as the mean ± sem. Data are representative of two independent experiments performed on 6 animals per group. **, *p* < 0.01, ***, *p* < 0.001, shown comparing the control group with the other groups; #, *p* < 0.05, ###, *p* < 0.001, shown comparing the RSV + LPS group with the LPS group; ^, *p* < 0.05, ^^^, *p* < 0.001, shown comparing the RSV + LPS group with the RSV group

Analysis for AHR showed that lung resistance (LR) of the RSV + LPS group was much higher than those of the control group, RSV group and the LPS group at methacholine concentrations of 6.25 mg/ml (all *P* < 0.05), 25 mg/ml (all *P* < 0.001) and 50 mg/ml (all *P* < 0.001) (Fig. 4).

### MMP-12 contributes to airway disorders provoked by LPS during the later stage of RSV infection

**Fig. 5:**
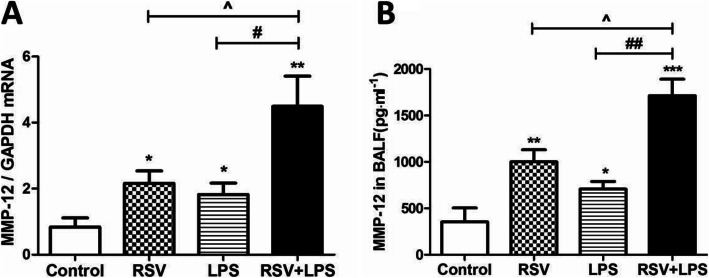
MMP-12 was markedly increased by LPS during the later stage of RSV infection. **a**: mRNA levels of MMP-12. **b**: MMP-12 protein in BALF. Values are expressed as mean **±** sem. Data are representative of two independent experiments performed on 6 animals per group. *, *p* < 0.05, **, *p* < 0.01, ***, *p* < 0.001, shown comparing the control group with the other groups; #, *p* < 0.05, ##, *p* < 0.01, shown comparing the RSV + LPS group with the LPS group; ^, *p* < 0.05, shown comparing the RSV + LPS group with the RSV group

**Fig. 6:**
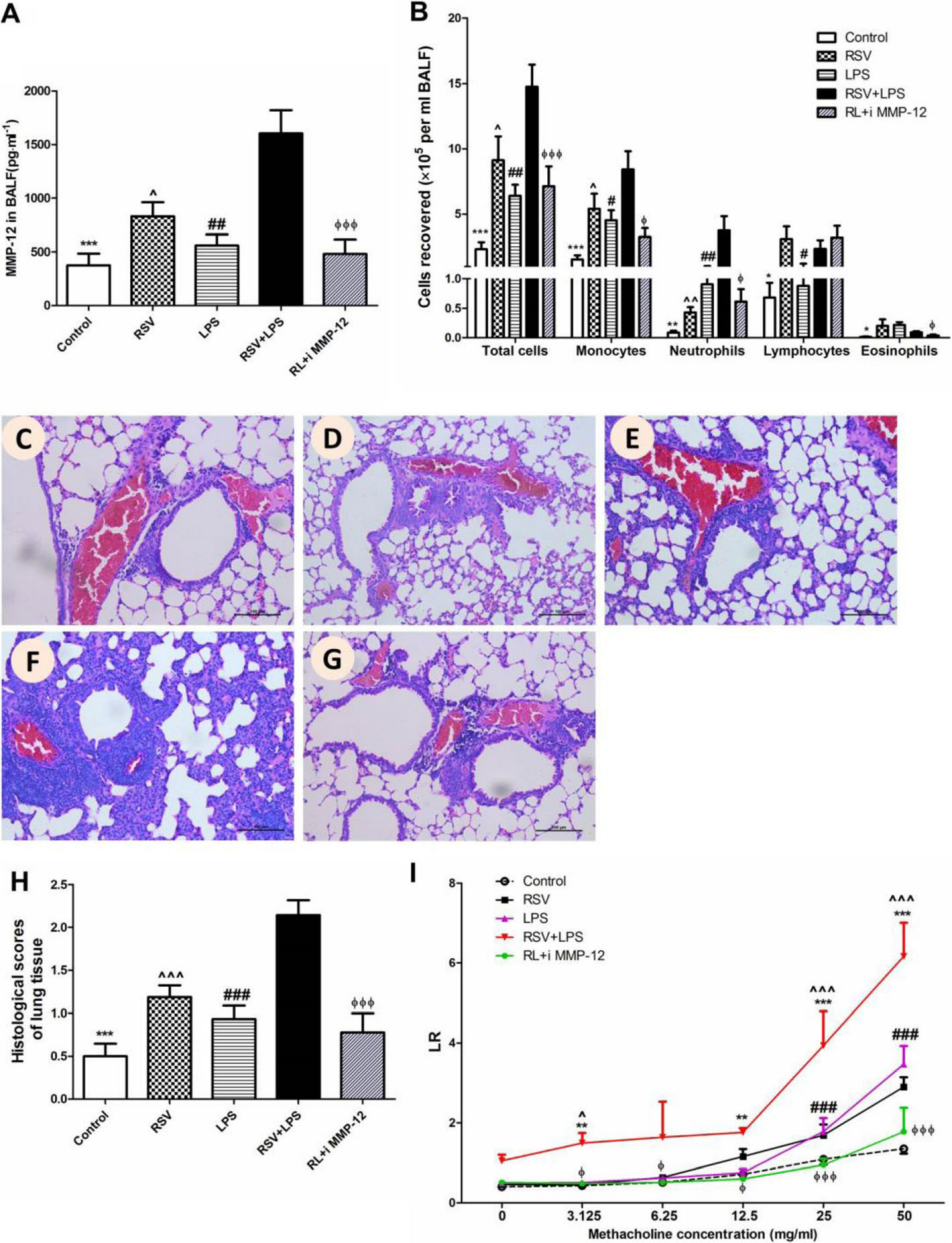
MMP-12 contributed to lung inflammation and AHR provoked by LPS during the later stage of RSV infection. MMP-12 levels (**a**), inflammatory cells in BALF (**b**), pathological damage of lung tissues (**c**-**h**) and AHR (**i**) were noteworthy alleviated by MMP-408. Representative HE staining: original Magnification × 200, **c**: control group; **d**: RSV group; **e**: LPS group; **f**: RSV + LPS group; **g**: RSV + LPS+ i MMP-12 group; **h**: Histological scores. Graphs are represented as the mean ± sem. Data are representative of two independent experiments performed on 6 animals per group. *, *p* < 0.05, **, *p* < 0.01, ***, *p* < 0.001, shown comparing the control group with the RSV + LPS group; #, *p* < 0.05, ##, *p* < 0.01, ###, *p* < 0.001, shown comparing the RSV + LPS group with the LPS group; ^, *p* < 0.05, ^^, *p* < 0.01, ^^^, *p* < 0.001, shown comparing the RSV + LPS group with the RSV group; ϕ, *p* < 0.05, ϕ ϕ, *p* < 0.01, ϕ ϕ ϕ, *p* < 0.001, shown comparing the RSV + LPS group with the RL + i MMP-12 group

Matrix metalloproteinase 12 (MMP-12) was involved in the pathogenesis of both RSV- and LPS- associated airway disorders. Thus, MMP-12 was detected. As shown in Fig. 5, both mRNA (A) and protein levels (B) of MMP-12 were much higher in the RSV + LPS group versus the other three groups (all *P* < 0.05). To further elucidate the deleterious role of MMP-12, MMP408, a specific inhibitor of MMP-12 was administrated to mice. As shown in Fig. 6a, MMP-12 was significantly blocked by MMP408 (all *P* < 0.05 for RSV + LPS group vs. the other groups). Synergistically, BALF inflammatory cells, monocytes, neutrophils, eosinophils (Fig. 6b, *P* < 0.05 for RSV + LPS group vs. the other groups) were all dramatically reduced in the MMP408-treated mice (RL + i MMP-12 group). Lung tissue damage (Fig. 6c-g), HPS (Fig. 6h, *P* < 0.001 for RSV + LPS group vs. the other groups) and AHR (Fig. 6i, *P* < 0.001 for RSV + LPS group vs. the other groups at methacholine concentrations of 25 mg/ml and 50 mg/ml) were all dramatically reduced in the RL + i MMP-12 group in parallel. Taken together, MMP-12 contributed to airway disorders provoked by LPS during the later stage of RSV infection.

### ERK signaling pathway is involved in MMP-12 production provoked by LPS during the later stage of RSV infection

**Fig. 7:**
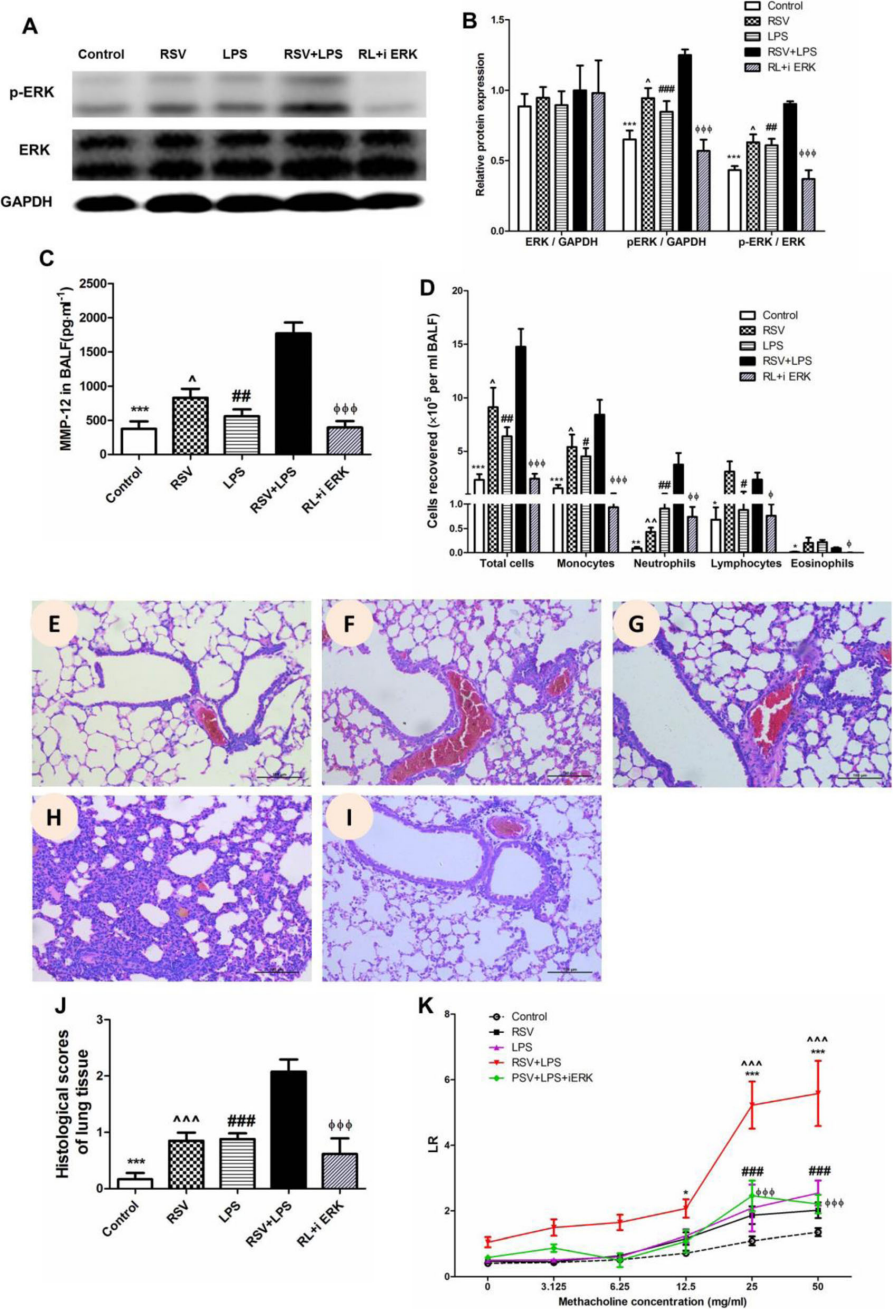
ERK signaling pathway contributed to MMP-12 over-production and the airway disorders provoked by LPS. Mice were treated intraperitoneally with the specific inhibitor of ERK. ERK expression was detected by western blot (**a**) and was semi-quantitatively assessed (**b**). MMP-12 levels (**c**), inflammatory cells (**d**), pathological damage of lung tissues (**e**-**i**), HPS (**j**) and AHR (**k**) were noteworthy alleviated by ERK inhibition. Representative HE staining: original Magnification × 200, **e**: control group; **f**: RSV group; **g**: LPS group; **h**: RSV + LPS group; **i**: RSV + LPS+ i ERK group; **j**: Histological scores. Graphs are represented as the mean ± sem. Data are representative of two independent experiments performed on 6 animals per group. *, *p* < 0.05, **, *p* < 0.01, ***, *p* < 0.001, shown comparing the control group with the RSV + LPS group; #, *p* < 0.05, ##, *p* < 0.01, ###, *p* < 0.001, shown comparing the RSV + LPS group with the LPS group; ^, *p* < 0.05, ^^, *p* < 0.01, ^^^, *p* < 0.001, shown comparing the RSV + LPS group with the RSV group; ϕ, *p* < 0.05, ϕ ϕ, *p* < 0.01, ϕ ϕ ϕ, *p* < 0.001, shown comparing the RSV + LPS group with the RL + i ERK group

MAPK signaling pathway has been reported to participate in MMPs production, so ERK, JNK and p38 were detected. As shown in Supplementary data 1, JNK (Supplementary data 1 A, B) and p38 (Supplementary data 1 C, D) were not increased in the RSV + LPS group. When JNK or p38 was inhibited respectively, MMP-12 levels were not decreased (Supplementary data 1 E). As shown in Fig. 7a and b, p-ERK was significantly induced in the RSV + LPS group (all *P* < 0.05 for RSV + LPS group vs. the other groups as to p-ERK semi-quantitative expression). To further examine the role of ERK pathway in MMP-12 modulation, a specific inhibitor of ERK (PD98059) was used, which truly decreased p-ERK (Fig. 6a-b). In parallel, MMP-12 levels (Fig. 7c, all *P* < 0.05 for RSV + LPS group vs. the other groups), BALF inflammatory cells, monocytes, neutrophils, lymphocytes, eosinophils (Fig. 7d, all *P* < 0.05 for RSV + LPS group vs. the other groups), lung tissue damage (Fig. 7e-i) and HPS (Fig. 7j, all *P* < 0.001 for RSV + LPS group vs. the other groups) and AHR (Fig. 7k, all *P* < 0.01 for RSV + LPS group vs. the other groups at methacholine concentrations of 25 mg/ml and 50 mg/ml), were all dramatically reduced in the ERK-inhibitor-treated mice (RL + i ERK group). Thus, MMP-12 provoked by LPS during the later stage of RSV infection was regulated by the ERK signaling pathway.

## Discussion

The results of this study clearly demonstrate that LPS exposure superimposed on RSV-infected lungs could lead to more vigorous cellular influx, lung structures damage, augmented AHR and higher MMP-12 levels. Inhibition of MMP-12 or ERK signaling pathway in vivo both diminished LPS-driven lung inflammation and AHR. Therefore, the mouse model we used in the current study shares key features with respiratory virus-induced exacerbation of asthma in humans, and a larger ERK-MMP-12 response observed in RSV-infected mice treated with LPS may account for the robust inflammatory cascade.

LPS is found ubiquitously in the environment, and on many occasions, it can create difficulties by inducing a large amount of inflammatory responses, even to the extent of shock or death of the host [19]. In asthmatic children, a significant correlation was found between levels of LPS and airway neutrophils in bronchoalveolar lavage [20]. In animal models, authors also identified that further LPS application in the airway might induce a state of worsened inflammation with neutrophilia predominant with eosinophilia persistence or abolished [21]. In our study, BALF and lung tissue inflammatory cells infiltration and AHR were markedly aggravated by LPS during the later stage of RSV infection. These results keep well consistent with the previous reports, emphasized the pathogenic roles of LPS. However, several epidemiological studies have contradictorily verified that exposure to environmental LPS in early childhood would reduce the incidence of allergic disorders in later life [22–24]. Animal studies also confirmed LPS exposure before or shortly after sensitization protects against the development of allergy [25]. Moreover, normal airway epithelial cells are relatively resistant to common type LPS stimulation [26]. Pretreated LPS protects the epithelial cells against the action of poly(I:C) and human parechovirus by attenuating TANK-binding kinase 1, IRF3, and NF- κB activation [17]. The disparity may be due to the difference of experimental animals, the timing of delivering LPS, the dose of LPS and the pathway (inhaled by aerosol in a chamber, intranasally or intravenously) [17, 21].

Previously, we have demonstrated that Th2 cytokines were triggered and accounted for the persistent airway inflammation induced by RSV [9]. Caballero MT. et al. [27] further revealed that interactions between TLR4 genotype and environmental LPS exposure modulated GATA3/T-bet ratios during RSV infection, and a Th2 bias with high levels of IL-4 increased disease severity in infants and murine models. Thus, in the present study, we first detected levels of IL-4, IL-5 and IL-13. Unexpectedly, as shown in Supplementary data 2, these cytokines were dramatically inhibited but not increased by LPS. The epithelia-derived cytokines TSLP, IL-25 and IL-33, which could promote asthmatic inflammation by driving Th2 responses [28, 29] were also suppressed by LPS. In addition, mRNA level of GATA-3 and ID2, the transcriptional factors related to Th2 cytokines were consistently decreased by LPS. Thus, other divergent pro-inflammatory mechanisms were indicated. LPS can promote a shift from Th2-derived airway eosinophilic inflammation to Th1 or Th17-derived neutrophilic inflammation in murine asthma models [30, 31]. However, the local switching of the airway inflammation from eosinophilia to neutrophilia did not quench the airway inflammation; instead, airway hyperreactivity was increased [27]. Accordingly, levels of IFN-γ, IL-10, IL-6, IL-17, IL-21 and IL-1β were subsequently assessed (Supplementary data 3). Again, none of these cytokines was increased following LPS treatment during the later stage of RSV infection.

The initial episodes of acute inflammation promote airway remodeling by altering the homeostasis of extracellular matrix in lungs. Matrix metalloproteinases (MMPs), characterized by mediating tissue remodeling and inflammatory processes, can help in maintaining homeostasis in basement membrane degeneration, epithelial repair and angiogenesis at the early phase of asthma exacerbation [32]. Several previous studies have shown that RSV triggers dramatic up-regulation of lung MMPs which can delay viral clearance and facilitate airway inflammation and AHR [33, 34]. We further identified MMP-12 as an important culprit in our RSV-infected nude mice and BALB/c mice models [10, 11]. Mebratu et al. [35] revealed that mice injured with elastase and LPS showed an enhanced and prolonged neutrophilic response to RSV that was associated with increased levels of IL-17and MMP-9. Therefore, levels of MMP-9 and MMP-12 were evaluated. MMP-9 was similarly markedly increased in both the RSV + LPS group and the LPS group (Supplementary data 4). MMP-12 was dramatically increased in the RSV + LPS group as compared to the RSV group and LPS group. Moreover, MMP-12 block substantially alleviated the airway disorders in the RSV + LPS mice. Hence, MMP-12 contributes to the exacerbated RSV-associated persistent lung inflammation and AHR caused by LPS treatment. The detrimental role of MMP-12 in the pathogenesis of COPD, emphysema, and asthma is also well established [36, 37].

LPS can activate multiple signaling cascades such as MAP kinases and recruitment of interleukin (IL)-1 receptor signaling complex, which involve Myd88 and IRAK [38]. In primary culture chondrocytes and cartilage explants, IL-1β could induce MMP-12 via MAP kinase signaling pathways [39]. We found that among the three MAPK proteins, p-ERK was significantly increased in the RSV + LPS group. When ERK was inhibited, MMP-12, lung inflammation and AHR were all correspondingly decreased. Xiao X et al. [40] reported that Oridonin, a diterpenoid compound extracted from traditional medicinal herbs, can inhibit the proliferation, invasion, and migration of gefitinib-resistant non-small cell lung cancer cells by suppressing EGFR/ERK/MMP-12 and CIP2A/PP2A/Akt signaling pathways. These observations are in support of our findings, suggesting that ERK signaling pathway is involved in LPS induced MMP-12 over-production during the later stage of RSV infection.

## Conclusions

In summary, the LPS-induced exacerbation of RSV-induced lung injury shares key features with respiratory virus-induced exacerbation of asthma in humans. Exposure to LPS in RSV-infected mice is associated with enhanced increases in ERK-MMP-12 expression that translates into increased lung inflammation and AHR. These findings contribute novel information to the field investigating the onset of post-RSV bronchiolitis recurrent wheezing as a result of LPS exposure.

## Supplementary information

**Additional file 1: Supplementary data 1**. JNK and p38 signaling pathway didn’t affect MMP-12 production induced by LPS during the later stage of RSV infection. Mice were treated intraperitoneally with the specific inhibitor of JNK or p38. JNK, p-JNK, p38 and p-p38 expressions were detected by western blot and were semi-quantitatively assessed (A-D). MMP-12 levels in BALF were assessed with ELISA (E). Data are representative of two independent experiments performed on 6 animals per group. ***, *p* < 0.001, shown comparing the control group with the RSV + LPS group; ##, *p* < 0.01, shown comparing the RSV + LPS group with the LPS group; ^, *p* < 0.05, shown comparing the RSV + LPS group with the RSV group; ϕ, *p* < 0.05, shown comparing the RSV + LPS group with the inhibitor-treated mice groups.

**Additional file 2: Supplementary data 2.** The Th2 responses were deduced by LPS during the later stage of RSV infection. Levels of IL-4 (A), IL-5 (B), IL-13 (C), TSLP (D), IL-25 (E), IL-33 (F) in BALF were detected with ELISA-based assays. mRNA levels of GATA-3 (G) and ID2 (H) were assessed with Q-PCR. Values are expressed as mean ± sem. Data are representative of two independent experiments performed on 6 animals per group. *, *p* < 0.05, **, *p* < 0.01, ***, p < 0.001, shown comparing the control group with the other groups; ^, p < 0.05, ^^, p < 0.01, ^^^, p < 0.001, shown comparing the RSV + LPS group with the RSV group.

**Additional file 3: Supplementary data 3.** The Th1 and Th17 cytokines in BALF were not provoked by LPS during the later stage of RSV infection. Levels of IFN-γ (A), IL-6 (B), IL-10 (C), IL-17A (D), IL-21 (E), IL-1β(F) in BALF were detected with ELISA-based assays. Values are expressed as mean ± sem. Data are representative of two independent experiments performed on 6 animals per group. *, p < 0.05, **, p < 0.01, shown comparing the control group with the other groups; ^, p < 0.05, shown comparing the RSV + LPS group with the RSV group.

**Additional file 4: Supplementary data 4**. The levels of MMP-9 in BALF. Values are expressed as mean ± sem. Data are representative of two independent experiments performed on 6 animals per group. *, p < 0.05, shown comparing the control group with the other groups; ^, p < 0.05, shown comparing the RSV + LPS group with the RSV group.

## Data Availability

All data generated or analysed during this study are included in this published article and its supplementary information files. The datasets used and/or analysed during the current study are available from the corresponding author on reasonable request.

## Abbreviations

AHR: Airway hyperresponsiveness
BALF: Bronchoalveolar lavage fluid
LRTI: Lower respiratory infection
LPS: Lipopolysaccharide
MMP-12: Matrix metalloproteinases-12
RSV: Respiratory syncytial virus
