# Supplementary figures and images for: LPS aggravates lung inflammation induced by RSV by promoting the ERK-MMP-12 signaling pathway in mice
Source: Respir Res. 2020 Jul 21;21:193. doi: 10.1186/s12931-020-01453-6 (PMC7372760; doi:10.1186/s12931-020-01453-6)

## Supplementary data 1

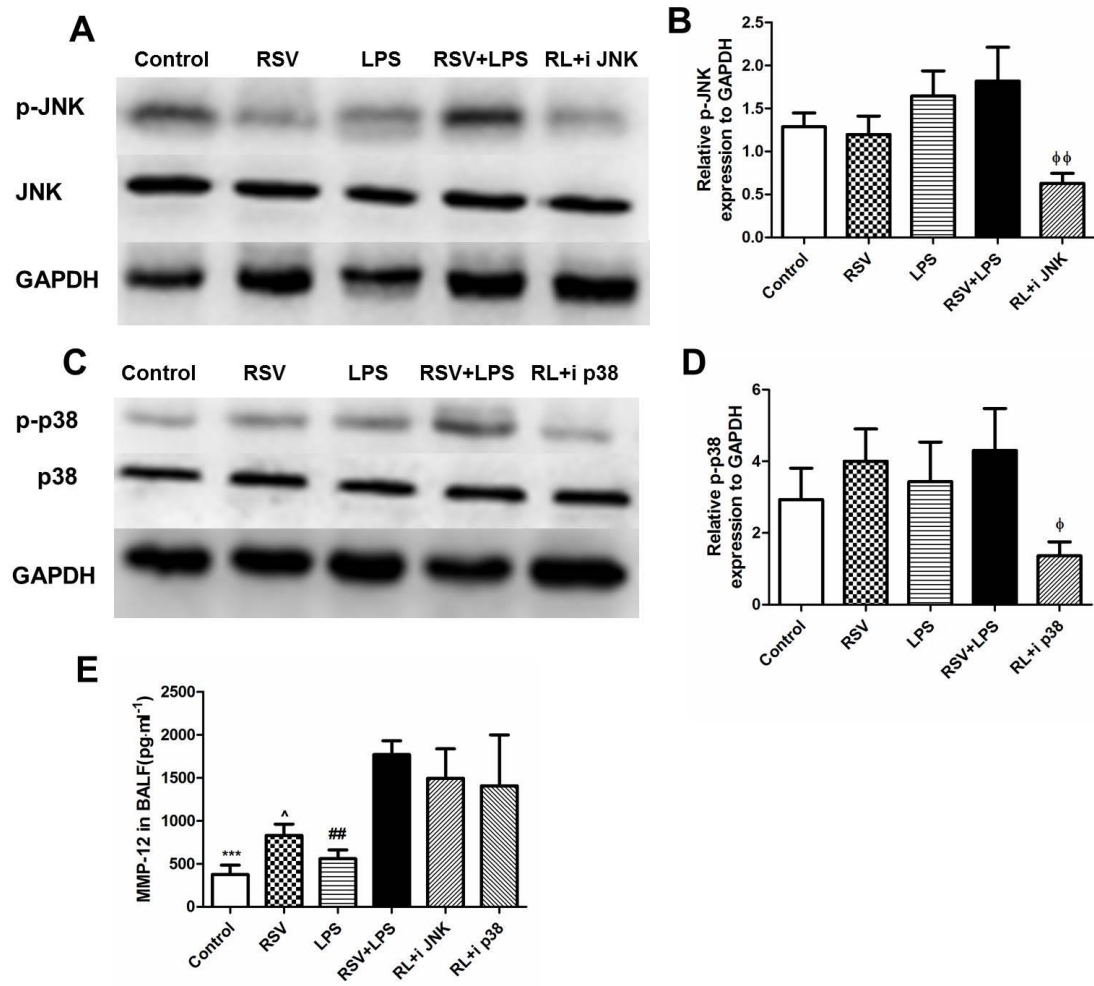

Supplement: Supplementary file 1 — Additional file 1: Supplementary data 1. JNK and p38 signaling pathway didn’t affect MMP-12 production induced by LPS during the later stage of RSV infection. Mice were treated intraperitoneally with the specific inhibitor of JNK or p38. JNK, p-JNK, p38 and p-p38 expressions were detected by western blot and were semi-quantitatively assessed (A-D). MMP-12 levels in BALF were assessed with ELISA (E). Data are representative of two independent experiments performed on 6 animals per group. ***, p < 0.001, shown comparing the control group with the RSV + LPS group; ##, p < 0.01, shown comparing the RSV + LPS group with the LPS group; ^, p < 0.05, shown comparing the RSV + LPS group with the RSV group; ϕ, p < 0.05, shown comparing the RSV + LPS group with the inhibitor-treated mice groups. [file 12931_2020_1453_MOESM1_ESM.pdf]

## Supplementary data 2

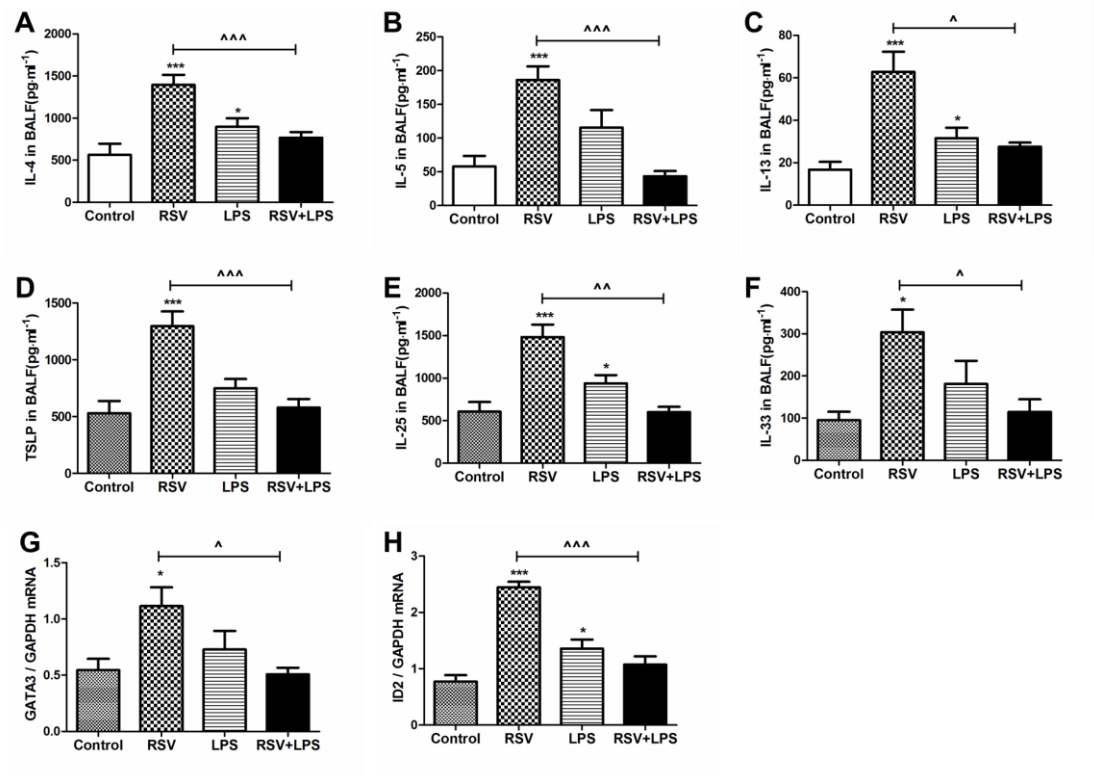

Supplement: Supplementary file 2 — Additional file 2: Supplementary data 2. The Th2 responses were deduced by LPS during the later stage of RSV infection. Levels of IL-4 (A), IL-5 (B), IL-13 (C), TSLP (D), IL-25 (E), IL-33 (F) in BALF were detected with ELISA-based assays. mRNA levels of GATA-3 (G) and ID2 (H) were assessed with Q-PCR. Values are expressed as mean ± sem. Data are representative of two independent experiments performed on 6 animals per group. *, p < 0.05, **, p < 0.01, ***, p < 0.001, shown comparing the control group with the other groups; ^, p < 0.05, ^^, p < 0.01, ^^^, p < 0.001, shown comparing the RSV + LPS group with the RSV group. [file 12931_2020_1453_MOESM2_ESM.pdf]

### Supplementary data 3

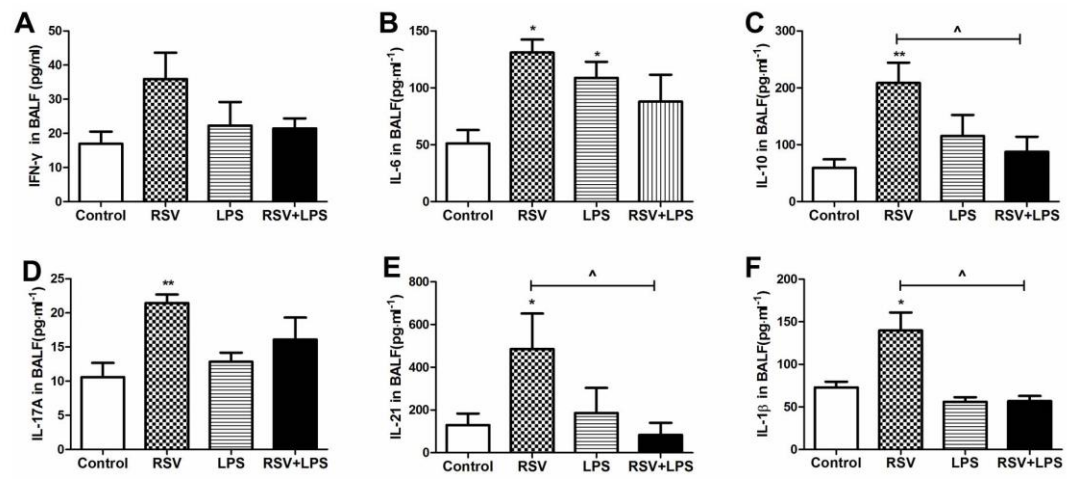

Supplement: Supplementary file 3 — Additional file 3: Supplementary data 3. The Th1 and Th17 cytokines in BALF were not provoked by LPS during the later stage of RSV infection. Levels of IFN-γ (A), IL-6 (B), IL-10 (C), IL-17A (D), IL-21 (E), IL-1β(F) in BALF were detected with ELISA-based assays. Values are expressed as mean ± sem. Data are representative of two independent experiments performed on 6 animals per group. *, p < 0.05, **, p < 0.01, shown comparing the control group with the other groups; ^, p < 0.05, shown comparing the RSV + LPS group with the RSV group. [file 12931_2020_1453_MOESM3_ESM.pdf]

#### Supplementary data 4

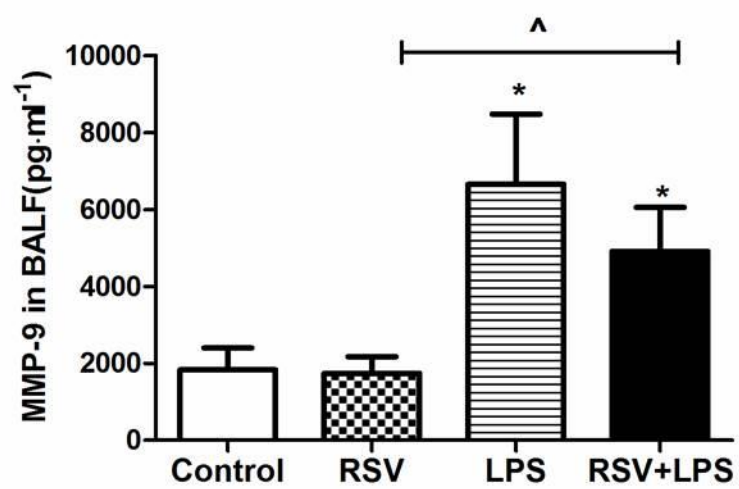

Supplement: Supplementary file 4 — Additional file 4: Supplementary data 4. The levels of MMP-9 in BALF. Values are expressed as mean ± sem. Data are representative of two independent experiments performed on 6 animals per group. *, p < 0.05, shown comparing the control group with the other groups; ^, p < 0.05, shown comparing the RSV + LPS group with the RSV group. [file 12931_2020_1453_MOESM4_ESM.pdf]
